# Benefits of Nut Consumption on Insulin Resistance and Cardiovascular Risk Factors: Multiple Potential Mechanisms of Actions

**DOI:** 10.3390/nu9111271

**Published:** 2017-11-22

**Authors:** Yoona Kim, Jennifer B. Keogh, Peter M. Clifton

**Affiliations:** School of Pharmacy and Medical Sciences, University of South Australia, General Post Office Box 2471, Adelaide, SA 5001, Australia; yoona.kim@unisa.edu.au (Y.K.); jennifer.keogh@unisa.edu.au (J.B.K.)

**Keywords:** nuts, type 2 diabetes mellitus, cardiovascular disease

## Abstract

Epidemiological and clinical studies have indicated that nut consumption could be a healthy dietary strategy to prevent and treat type 2 diabetes (T2DM) and related cardiovascular disease (CVD). The objective of this review is to examine the potential mechanisms of action of nuts addressing effects on glycemic control, weight management, energy balance, appetite, gut microbiota modification, lipid metabolism, oxidative stress, inflammation, endothelial function and blood pressure with a focus on data from both animal and human studies. The favourable effects of nuts could be explained by the unique nutrient composition and bioactive compounds in nuts. Unsaturated fatty acids (monounsaturated fatty acids and polyunsaturated fatty acids) present in nuts may play a role in glucose control and appetite suppression. Fiber and polyphenols in nuts may also have an anti-diabetic effect by altering gut microbiota. Nuts lower serum cholesterol by reduced cholesterol absorption, inhibition of HMG-CoA reductase and increased bile acid production by stimulation of 7-α hydroxylase. Arginine and magnesium improve inflammation, oxidative stress, endothelial function and blood pressure. In conclusion, nuts contain compounds that favourably influence glucose homeostasis, weight control and vascular health. Further investigations are required to identify the most important mechanisms by which nuts decrease the risk of T2DM and CVD.

## 1. Introduction

The prevalence of diabetes mellitus will increase worldwide from 382 million individuals in 2013 to 592 million individuals in 2035. Type 2 diabetes mellitus (T2DM) accounts for 90–95% of individuals with diabetes mellitus [1]. T2DM is attributable to poor diet, obesity, age, genetics, smoking, sedentary lifestyle and hypertension [2,3]. T2DM is associated with an increased risk of adverse cardiovascular events [3]. A 1 mmol/L increase in fasting plasma glucose is related to a 17% increase in the risk of developing of cardiovascular disease (CVD) and death [4]. Individuals with diabetes have a 2- to 4-fold higher risk of CVD than individuals without diabetes [5]. As a consequence, T2DM dramatically increases health care costs and disease burden [6]. Lifestyle modification including diet can decrease the risk of developing T2DM [7]. A large number of epidemiological and clinical studies have shown that consumption of different types of nuts (almonds, walnuts, hazelnuts, pecans, pistachios, macadamia nuts, cashews and Brazil nuts) is associated with a reduced risk of T2DM [8,9,10] and CVD [8,9,10,11,12]. 

This review aims to address the potential mechanisms of nut consumption on the prevention and treatment of T2DM and CVD, focusing on body weight control, glucose control, gut microbiota composition, inflammation, oxidative stress, lipid profiles, endothelial function and hypertension. All human interventions available on PubMed that covered these areas were included. This is the only systematic review that covers all these areas.

## 2. Nut Composition

Nuts are an energy dense food (Table 1). Nuts consist of fat (43–67% by weight), protein (8–22%), ash (1–3%), total soluble sugars (0.6–4%), polyphenols (0.2–1.6%), phytates (0.2–0.4%) [13]. Nuts also contain bioactive components such as lipids (carotenoids and phytosterols), vitamins and minerals (particularly magnesium, folate and potassium).

Nuts predominantly contain unsaturated fatty acids (monounsaturated fatty acids (MUFAs) and polyunsaturated fatty acids (PUFAs)) with a low amount of saturated fatty acids (SFAs—only 4–5%). Oleic acid (C18:1) is the major MUFA in all nuts [13]. Linoleic acid (C18:2*n*-6) is the major PUFA in nuts except for macadamias [13]. Walnuts have the highest content of PUFAs at 47%, 38% linoleic acid and 9% linolenic acid (C18:3*n*-3). Nuts are also a good source of polyphenols and fiber (especially nuts covered with skin). In the US diet in 2008, nuts provided 162 mg/day of polyphenols (19%) compared with 204 mg/day from vegetables (24%), 213 mg/day from grains (25%) and 223 mg/day from fruits (26%), with similar figures in Europe [14]. Pistachios contain the highest quantity of lutein and zeaxanthin which are xanthophyll carotenoids. These carotenoids appear to protect from age-related macular degeneration [15].

## 3. Glycemic Control

A meta-analysis of 12 randomised controlled trials (RCTs) with a ≥3-week follow-up period in subjects with T2DM comparing a diet supplemented with tree nuts (almonds, Brazil nuts, cashews, hazelnuts, macadamia nuts, pecans, pine nuts, pistachios and walnuts) and an isocaloric diet without tree nuts, showed that consumption of tree nuts at a median dose of 56 g/day improved glycemic control in subjects with T2DM, showing significantly decreased glycosylated haemoglobin levels (HbA1c; mean difference 0.07%, *p* = 0.0003) and fasting glucose (*p* = 0.03), with no effect on fasting insulin or homeostasis model assessment of insulin resistance index (HOMA-IR). Most RCTs were of poor quality and had short study periods [20]. Human interventions evaluating the effect of nut consumption on glycemic control are shown in Table 2. In summary, given the results from the meta-analysis of 12 RCTs [20] and chronic studies [21,22,23,24,25,26] that were not included in the meta-analysis [20], nut consumption benefits glycemic control regardless of the type of nut but the effect is small and the amount of nuts required is large. Moreover, nut consumption reduces postprandial glycemia responses as shown in all nine studies [27,28,29,30,31,32,33,34,35]. 

A randomized control clinical trial in subjects with T2DM showed that consumption of walnut oil (15 g/day for 3 months) significantly reduced glycosylated HbA1c by 8 ± 22% (*p* = 0.005) and fasting glucose levels by 8 ± 17% (*p* = 0.001) compared with the baseline while a control group showed no significant differences in HbA1c and fasting glucose levels. [21].

Pistachio consumption improved glycemic status in subjects with T2DM [22,23] and in healthy young men [24]. In the 8-week dietary intervention trial [24], healthy young men aged on average 22 years consumed a Mediterranean diet for 4 weeks and in the next 4 weeks they supplemented the Mediterranean diet with pistachios for 4 weeks replacing 32% of the energy obtained from MUFAs in the control diet (20% of energy). A Mediterranean diet rich in pistachios improved glucose levels (−8.8 ± 8.5% *p* < 0.001), low-density lipoprotein cholesterol (LDL-C; *p* < 0.001), total cholesterol (TC; *p* < 0.001), triacylglycerol (*p* = 0.008), TC-C/high density lipoprotein cholesterol (HDL-C) ratio (*p* < 0.001), LDL-C/HDL-C ratio (*p* < 0.001) and endothelium-dependent vasodilation (*p* = 0.002; 30% relative increase). In addition, the pistachio diet significantly decreased serum oxidation (lipid hydroperoxide (*p* < 0.001) and malondialdehyde (MDA; *p* < 0.001)) and inflammatory markers (interleukin-6 (IL-6; *p* < 0.001)) and increased superoxide dismutase (SOD—a superoxide anion radical (O^2−^) scavenger; *p* < 0.001) in comparison with a Mediterranean diet rich in vegetable and fish and limited in red meat, fat and egg products [24].

Almond consumption improved glucose control in subjects with impaired fasting glucose [25] and prediabetes [36], while one study showed no effect of an almond diet on glucose and lipid profiles in subjects with T2DM [37]. In a randomized parallel trial [36], subjects with prediabetes who followed an ADA (American Diabetes Association) diet containing 20% of energy from almonds (60 g/day of pre-packaged raw or dry roasted almonds) for 16 weeks showed a significant reduction in fasting insulin concentrations (−23% vs. +19%; *p* = 0.002), HOMA-IR (−25% vs. +0.3%; *p* = 0.007) and homeostasis model analysis for beta-cell function (HOMA-B; −18% vs. +30.0%; *p* = 0.001) with no alteration in fasting glucose, compared with an almond-free control group. LDL-C concentrations were significantly lowered after the almond diet compared with the control diet containing 60–70% carbohydrate and MUFA together, 15–20% protein, 10% saturated fat, and 300 mg/day cholesterol. Adjustment for weight did not abolish changes in insulin, HOMA-IR, and HOMA-B. Subjects in the almond group were instructed to use the ADA Food Exchange System for 80% of their energy needs. The average body mass index (BMI) was 30 ± 5 kg/m^2^ for the almond group (*n* = 32) and 29 ± 5 kg/m^2^ for the control group (*n* = 33). Energy intake was limited for 14 subjects with a BMI > 25 kg/m^2^ out of 65 subjects following the ADA’s recommendations of modest weight loss in people with prediabetes. No significant differences in weight, BMI and waist circumference were observed between two groups at weeks 0, 4, 8, 12, and 16, even though the reductions in these measurements occurred in both groups over the course of the study (the average weight loss was 1.1 kg for the almond group and 2.0 kg for the control group) [36]. 

The negative study [37], which was included in the meta-analysis of T2DM subjects [20], consisted of two studies to assess insulin sensitivity. Both studies showed no effect on insulin sensitivity in healthy subjects or on glycemic control in subjects with T2DM, but beneficial effects were seen on lipid profiles. In study 1, 20 healthy adults were instructed to adhere to their habitual diet supplemented with 100 g almonds/day for 4 weeks. No changes in fasting glucose and fasting insulin, and insulin sensitivity index (SI) and glucose effectiveness (SG) as calculated using the Minimal Model method were observed before and after the diet. Body weight significantly increased (*p* = 0.006) and a significant time-by-sex interaction for SI and SG was observed due to an increase in women with a decrease in men. Changes in body weight had no effect on insulin sensitivity. In study 2 using a randomised, double-blind, crossover design, volunteers were randomly assigned to begin one of four diets for 4 weeks: high-fat, high-almond (37% total fat, 10% from almonds); low-fat, high-almond (25% total fat, 10% from almonds); high-fat control (37% total fat, 10% from olive oil or canola oil); low-fat control (25% total fat, 10% from olive or canola oil). The almond diets contained 57–113 g almonds/day based on the total energy requirements. No changes in fasting glucose, fasting insulin, and 2-h glucose and insulin as assessed by oral glucose tolerance test were observed overall with almonds, while a significant interaction between fat source and fat level for both fasting and 2-h glucose levels was observed due to the lower glucose levels on the low fat, high almond diet than a high fat control diet [37].

### 3.1. Acute Studies

A favourable effect of pistachios on postprandial glycemia was seen in 10 healthy adults [27] and 20 subjects with metabolic syndrome [28]. Two acute meal studies [29,30] examined the glucose-lowering effect of peanuts. Peanut consumption significantly reduced the 60-min postprandial glucose response to high-glycemic load meals in healthy subjects [29,30]. Five acute meal studies with a crossover design examined the effect of almond intake on postprandial glycemia and insulinemia [31,32,33,34,38]. Almonds (28 g) with a test meal (bagel, juice, and butter) significantly lowered postprandial glycemia in seven subjects with T2DM by 30% (*p* = 0.04), but not in 13 healthy subjects without T2DM, with no differences in insulinemia and the incretin hormone, glucagon-like peptide-1 (GLP-1) [38]. On the other hand, four acute meal studies showed attenuated postprandial glycemia in impaired glucose tolerant [31], healthy [32,33,35] and diabetic [35] subjects. 

### 3.2. Possible Nutrients Involved in Glucose-Lowering Effects in Nuts

A recent meta-analysis of 102 RCTs showed the substitution of carbohydrates and SFAs with a diet rich in unsaturated fat, particularly PUFAs improved glucose control. The substitution of carbohydrates with PUFAs significantly decreased HbA1c (−0.11%; 95% CI: −0.17, −0.05) and fasting insulin (−1.6 pmol/L; 95% CI: −2.8, −0.4). Substitution of carbohydrates with MUFAs decreased HbA1c (−0.09%; 95% CI: −0.12, −0.05), 2-h postprandial insulin (−20 pmol/L/min; 95% CI: −32, −8), and HOMA-IR (−2%; 95% CI: −5, −0.3). The substitution of SFAs with MUFAs significantly lowered HbA1c (−0.12%; 95% CI: −0.19, −0.05) and HOMA-IR (−3%; 95% CI: −6, −0.4), while the substitution of SFAs with PUFAs significantly lowered HbA1c (−0.15%; 95% CI: −0.2, −0.06), C-peptide (−0.07%; 95% CI: −0.14, −0.01), HOMA-IR (−4%; 95% CI: −6, −2) and 2-h postprandial insulin responses (0.5 pmol/L/min; 95% CI: 0.2, 0.8) [39]. Alpha-linolenic acid (ALA an-3 PUFA; 18:3*n*-3; a precursor of eicosapentaenoic acid (EPA; 20:5*n*-3) and docosahexaenoic acid (DHA; 22:6*n*-3) has been shown to reduce fasting glucose levels in a meta-analysis of 12 RCTs with a ≥4-week intervention and a ≥4-week washout period [40] and to decrease the prevalence of insulin resistance in middle-aged Japanese but only in those with a normal weight [41]. ALA stimulates GLP-1 release from pancreatic β-cells [42,43], from intestinal l-cells [42] through activation of GPR40, one of the G-protein-coupled free fatty acid receptors also called free fatty acid receptor 1 (FFAR1). In addition, ALA induces insulin-like growth factor 1 (IGF-1) gene expression and release from hepatocytes via a peroxisome proliferator-activated receptor α (PPAR α) dependent pathway [44,45] resulting in improved insulin action primarily in skeletal muscle, and in adipose tissue [45].

In summary, unsaturated fatty acids in nuts in place of both carbohydrate and saturated fat appear to improve glucose levels by a small amount but more studies at the molecular level should be undertaken along with a detailed investigation of different nut types and amounts, study duration, background diet, subject ethnicity in subjects with T2DM and at risk of T2DM. 

### 3.3. Modification in Micro RNAs Related to Insulin Sensitivity

Only two nut intervention studies have explored microRNA modification in relation to T2DM risk [46,47]. Ortegaa et al. [46] found significant modification of several common miRNAs in plasma from 10 healthy women who consumed a PUFA-enriched normocaloric diet containing 15 g/day of almonds and 15 g/day of walnuts (55–60% carbohydrates 15% proteins, 30% fat, 10% SFA, 10–15% MUFAs and 10% PUFAs) for 8 weeks. The changes of plasma miR-106a were correlated with changes in circulating PUFAs. The changes in plasma miR-130b and miR-221 (*r* = 0.46, *p* = 0.03) were correlated with changes in plasma C-reactive protein. The changes in plasma miR-125a-5p were correlated with changes in plasma fasting triglycerides and adiponectin [46]. Very recently, a 4-month diet supplemented with 57 g/day of pistachio (50% carbohydrates, 33% fat) modified circulating microRNAs (miR-192 and miR-375) and these changes were positively correlated with plasma glucose, insulin and HOMA-IR in 49 subjects with prediabetes [47]. Thus, microRNAs can be modulated by nut consumption and may be involved in glucose metabolism, lipid metabolism and inflammation. Circulating miRNAs have stable and reproducible levels in serum and may be useful biomarkers examining molecular mechanisms in T2DM and CVD [48].

## 4. Body Weight Control, Appetite, Energy Balance and Lipid Bioaccessibility

Nuts may be an effective appetite suppressant to prevent body weight gain. In addition, nuts may enhance energy expenditure and reduce energy absorption [37,49,50,51,52,53,54].

### 4.1. Body Weight Control

The high energy density and fat content of nuts has raised concerns that regular nut consumption will cause body weight gain. However, epidemiological studies indicated either an inverse [55,56,57,58] or no association [59,60,61] between nut consumption and BMI or body fat level. Most randomised crossover interventions investigating the effect of adding nuts to a habitual diet on body weight as a primary endpoint reported either no weight gain or lower weight gain than expected from the additional energy intake from the nut consumption (peanuts [49], almonds [37,50,51,53,62,63,64,65], walnuts [52,53,66,67,68,69], pecans [70], pistachios [71,72,73,74], macadamia nuts [75,76] and hazelnuts [77]). 

Human interventions evaluating the effect of nut consumption on body weight control are shown in Table 3. Seventeen of 21 clinical studies in Table 3 showed no change in body weight but five [37,49,50,66,77] of 21 studies showed body weight gain ranging from 0.4 to 1 kg regardless of the types of nuts. Four studies [26,78,79,80] showed body weight reduction.

The PREDIMED study showed that adherence to the Mediterranean diets enriched with either 50 mL/day (350 mL/week) of extra virgin olive oil or 30 g/day of nuts for 3 years resulted in a reduction in body weight. The increased energy density was not associated with body weight gain [78]. Other short-term interventions also showed a greater weight loss effect of a diet enriched in nuts in comparison with a low-fat diet [79,80], or a calorie-matched complex carbohydrate diet [26]. In a randomized controlled trial comparing the effect of a moderate fat low energy diet (fat: 35% of energy; predominantly PUFAs), with a low-fat, low-energy diet (fat: 20% of energy) on weight loss in 101 overweight subjects for 18 months, the nut-enriched diet resulted in greater sustained weight reduction at 18 months compared with a low-fat diet [79]. Supplementation with either a high dose (70 g/day) or a recommended dose of pistachio nuts (42 g/day) for 12 weeks in a parallel design did not alter BMI or waist-to-hip ratio compared with a control diet in Chinese subjects with metabolic syndrome [73]. 

In summary, it would appear that nuts can be added to a diet without significant body weight gain under most circumstances.

### 4.2. Appetite

Two studies showed a favourable effect on appetite of almond snacks in subjects at increased risk of T2DM [81] and in healthy women [82]. In a 4-week randomized, parallel-arm study, 43 g almonds/day with breakfast or lunch, or alone as a morning or afternoon snack for 4 weeks, attenuated postprandial glucose responses and decreased hunger and desire to eat in subjects with increased risk for T2DM [81].

Cholecystokinin (CCK), GLP-1 and peptide YY (PYY) are gut satiety hormones. Cholecystokinin (CCK-8) is released from duodenal enteroendocrine cells in response to fatty acid and protein. GLP-1 and PYY are secreted from the ileum in response to carbohydrates, proteins and fatty acids. Ghrelin is also a gut hunger hormone released from the stomach which is suppressed by food intake. It increases appetite [83,84].

A study consisting of an in vitro (STC-1 cell–intestinal secretin tumor cell line) study and a human intervention suggested Korean pine nuts containing ≥92% PUFA (15% pinolenic acid (C18:3), linoleic acid (C18:2) and MUFAs (oleic acid (C18:1)) might be a good appetite suppressant (assessed by decreased prospective food intake) by enhancement of CCK-8 and GLP-1 secretion [85].

A favourable acute and second meal effect of added nuts was seen with almonds ingested by subjects with impaired glucose tolerance [31] and peanuts consumed by obese women with a high T2DM risk [29] with breakfast on postprandial glucose responses and appetite. 

Leptin is a hormone secreted from adipocytes which regulates energy balance by suppressing appetite along with other metabolic effects. Leptin plays an opposite role to that of ghrelin [86]. A recent meta-analysis of 20 RCTs showed that nut consumption (tree nut, peanut, and soy nut) significantly reduced leptin but did not significantly alter high sensitivity C-reactive protein (hs-CRP), IL-6, adiponectin, IL-10, and tumor necrosis factor-α (TNF-α) [87]. 

The physical texture of nuts may also affect satiety through the enhanced mastication required [88,89].

### 4.3. Energy Balance

Minimal [37,49,50,66,77] or no body weight gain [51,52,53,62,63,64,65,67,68,69,70,71,72,73,75,76] following nut consumption may also be due to increased energy expenditure. Several studies [49,54,90] have reported an increased resting metabolic rate which could be attributed to both the high unsaturated fat in nuts as well as the protein. A Mediterranean diet supplemented with nuts decreased waist circumference after 4.8 years of follow-up compared with a low-fat diet [91].

### 4.4. Bioaccessibility of Nutrients 

Bioaccessibility refers to the proportion of nutrients released from a complex food matrix, which become potentially available for absorption in the gastrointestinal tract [92]. One proposed mechanism of low or no weight gain with nuts includes a reduced level of lipid bioaccessibility from nuts. The properties of cell walls and fibre-rich skins in nuts may play a role in the rate and extent of lipid release [93].

Several studies [93,94] have investigated this question. In the first study [93], only the first layer of cells at the fracture surface was ruptured by chewing and released lipids. In faecal samples from healthy subjects who consumed the almond rich diet, intact almond tissues, intact cotyledon cells containing intracellular lipid, increased lipid concentrations, and large numbers of bacteria adherent to the cotyledon cells were observed [93]. Cell walls of almonds consist of non-starch polysaccharides (especially, arabinose-rich polysaccharides) and phenolic compounds (predominantly, protocatechuic acid, p-hydroxybenzoic acid, and vanillic acid).

Other intervention studies also showed an increased excretion of stool fat after consumption of almonds [51,88,95], pecans [96] and peanuts [97,98].

The poor lipid bioaccessibility of almonds reduces postprandial lipidemia which may be associated with a lower risk of coronary heart disease [99,100,101]. A randomised crossover study [102] in healthy human subjects compared 54 g fat in four different meals (whole almond seed, almond oil and defatted almond flour, or a sunflower oil blend), and showed a favourable effect of whole almond seed on postprandial lipidemia compared with other forms of lipid. However, no significant differences in insulin, 8-isoprostane F2 α (a marker of oxidative stress) and peripheral augmentation index (a measure of vascular tone) were seen between meals. The iAUC for plasma glucose, however, was higher on the whole almond seed meal than on the almond oil and defatted almond flour meal [102]. 

## 5. Gut Microbiota Modification

Fiber and polyphenols (flavonoids and nonflavonoids) abundant in nuts may exert a prebiotic effect and influence glucose metabolism [103]. Nuts with the highest contents of polymerized polyphenols are hazelnuts and pecans followed by pistachios, almonds and walnuts [104]. Walnuts, pecans and chestnuts are high in ellagitannins (hydrolysable tannins), whereas proanthocyanidins (condensed tannins) are abundant in the kernel of nuts such as hazelnuts, pecans, pistachios, almonds and cashew [19]. 

Proanthocyanidins are oligomers or polymers of polyhydroxy flavan-3-ol units, such as (−)-epicatechin, and (+)-catechin [105]. Epidemiological studies have shown an inverse association between proanthocyanidin intake and incident T2DM [106,107]. No dietary intervention examining the prebiotic effects of nut-derived proanthocyanidin has been performed while two dietary interventions have shown a favourable prebiotic effect of proanthocyanidin derived from grape seeds [108] and cocoa [109] modifying the proportion of *Bifidobacterium* and *Enterobacteriaceae* [108,109] which was not dose related. The doses of proanthocyanidins administered were 190 mg/day for healthy adults aged 40–59 years for 2 weeks [108], 380 mg/day for elderly adults aged 67–98 years for 2 weeks [108], and 494 mg/day for young healthy adults with a mean age of 30 years for 4 weeks [109]. 

### 5.1. Urolithin (Microflora Metabolite)

Walnuts and pecans which have the highest total phenol contents are the richest sources of ellagic acid that possesses 2,2-diphenyl-1-picrylhydrazyl (DPPH•) scavenging capacities [19]. Brazil nuts contain no polyphenols nor have an antioxidant capacity [19]. Ellagitannins and ellagic acid are not absorbed in the gastrointestinal tract but are metabolized by the human gut microbiota to urolithins (dibenzo[*b,d*]pyran-6-one derivatives with different hydroxyl substitutions) which are much better absorbed than the parent ellagitannins and ellagic acid [110] while the type of urolithin formed depends on particular species in the gut microflora [111]. There is a wide range of urolithins based on a decreasing number of phenolic hydroxyl groups; urolithin D→C→A→B [112]. Aromatic and phenolic structures including phenylvalerolactones and phenylvaleric, phenylpropionic, phenylacetic, hippuric, and benzoic acids, with different hydroxylation patterns which are microbial metabolites of flavan-3-ol dimers, oligomers, flavan-3-ol polymers [113,114,115,116]. Hydroxyphenylvalerolactones in plasma or urine are proposed to be the main metabolites of flavan-3-ol compounds as they come from reactions of the flavonoid C-ring-opening followed by lactonization [113,114]. In vitro and in vivo studies have shown beneficial effects of urolithins on oxidation, inflammation and glycation [110,117]. 

In a randomized parallel intervention in subjects with the metabolic syndrome comparing a 12-week healthy diet supplemented with 30 g/day mixed nuts (15 g walnuts, 7.5 g almonds and 7.5 g hazelnuts) with a healthy diet without nuts [118,119,120,121], a healthy diet with added nuts significantly increased walnut ellagitannin-derived urolithins A and B in urine [118,119], significantly improved fasting insulin, insulin sensitivity assessed by HOMA-IR, and IL-6 and significantly decreased DNA damage assessed by urinary 8-oxo-7,8-dihydro-2′-deoxyguanosine (a measure of oxidative stress) [121]. Adjustment for weight loss attenuated the statistical significance of IL-6 (*p* = 0.08) [120]. No effects were seen on cholesterol levels [120], biomarkers of oxidative stress (plasma oxidized LDL, plasma conjugated diene and urinary 8-isoprostanes), antioxidant capacity or endothelial function determined by non-invasive peripheral artery tonometry [121]. 

In a study with a metabolomic approach, nut consumption resulted in increased metabolism and excretion of unsaturated fatty acids with increased detection of fatty acid conjugated metabolites and increased excretion of serotonin metabolites in 24-h urine samples [118]. 

In summary, urolithins are phenolic metabolites which are increased following nut consumption, but to our knowledge, no human intervention study has investigated the prebiotic effect of nut derived urolithins. Therefore, it is unclear what role urolithins play in decreasing the risk of T2DM. 

### 5.2. Butyrate (Microflora Metabolite)

A few studies have examined the effect of nut consumption on gut microbiota and the gut metabolome [122,123,124,125]. In vitro, chestnut extract enhanced the viability of *Lactobacillu*s strains in a simulated GI tract [122]. In vitro using a model stomach, small intestine and colon finely ground almond seeds significantly increased the growth of *bifidobacteria* and *Eubacterium rectale* with a butyrogenic prebiotic effect [123].

Butyrate is a short chain fatty acid (SCFAs). SCFAs stimulate the expression of peptide YY via the G-protein-coupled receptors (Gpr41 and Gpr43) leading to the inhibition of gut motility and suppression of appetite [126,127]. Activation of G-protein-coupled receptors (Gpr43; and Gpr41) on l-cells by SCFAs triggers the secretion of GLP-1 which improve glucose homeostasis by increasing insulin and decreasing glucagon secretion [128,129]. SCFAs play a role in controlling body energy utilization by suppressing insulin signalling in adipocytes and fat accumulation in adipose tissues through Gpr43 activation [130]. Intestinal gluconeogenesis (IGN) may have a favourable impact on the risk of T2DM by decreasing food intake, body weight, hepatic glucose production and plasma glucose [131,132,133]. Butyrate promotes IGN via cyclic adenosine monophosphate (cAMP) in a Gpr41-independent manner, while propionate (a longer SCFA) promotes IGN via a gut-brain neuronal circuit by binding to Gpr41 [134]. 

Anti-inflammatory effects of butyrate have been demonstrated through two pathways: nuclear factor-κB (NF-κB) and histone deacetylase (HDAC) inhibition [135,136]. Butyrate suppresses the expression of inducible nitric oxide synthase (iNOS), IL-6 and TNF-α by downregulating NF-κB and extracellular signal-regulated kinase (ERK) signalling pathways in part with no involvement of the Jak/STAT pathway in IFN-γ induced-RAW 264.7 murine macrophage cells [137]. Butyrate also acts as a histone deacetylase (HDAC) inhibitor [135,136,138,139]. HDAC inhibitors could be a potential T2DM therapy [140,141]. HDACs inhibit the expression of glucose transporter type 4 (GLUT4) leading to decreased insulin sensitivity in adipocytes and muscle cells [140,141]. In the liver, HDACs stimulate forkhead box O (FoxO) DNA-binding leading to facilitated gluconeogenetic gene expression (e.g., glucose-6-phosphatase (G6Pase) and phosphoenolpyruvate carboxykinase (PEPCK)) [142], and HDACs act on a signal transducer and activator of transcription 3 (STAT3)-mediated gluconeogenesis [143]. In pancreatic β-cells, HDAC1 reduced the expression of duodenal homeobox 1 (PDX1) leading to a decreased expression of insulin [144,145]. Butyrate also induced regulatory T cells (Treg) that contribute to shutting down inflammatory responses, possibly by enhancing histone H3 acetylation and stimulating the expression of the transcription factor forkhead box P3 (FOXP3) in TReg cells [146,147,148,149]. In addition, butyrate attenuates MCP-1 levels in human PBMC [150] and represses the expression of vascular cell adhesion molecule-1 (VCAM-1) in human umbilical vein endothelial cells (HUVEC) [151,152].

Ukhanova et al. [124] conducted two separate randomised, controlled, crossover nut feeding studies in healthy subjects (*n* = 18 for an almond group; *n* = 16 for a pistachio group) who consumed 0, 43 g and 86 g per day for 18 days. Analyses of faecal samples using a 16S rRNA-based approach showed that nuts altered the composition of the faecal bacterial and fungal microbiota with an increase in butyrate producing bacteria. This effect was much stronger in the pistachio group. Neither nut altered *Lactobacillus* or *Bifidobacteria* levels [124]. An intervention with a much longer period (6 weeks) in healthy subjects indicated that whole almond or almond skin could beneficially modify gut microbiota and bacterial activities [125]. In this study [125], daily consumption of 56 g roasted, unsaltednwhole almonds or 10 g almond skin significantly increased populations of *Bifidobacterium* spp. and *Lactobacillus* spp., and suppressed the growth of *Clostridium perfringens*, leading to changes in bacterial enzyme activities (increased faecal β-galactosidase and decreased faecal β-glucuronidase, nitroreductase and azoreductase) [125].

In summary, butyrate is a major product of a nut-related change in the microbiota. Butyrate appears to be a strong candidate molecule which can be mechanistically linked to a reduced T2DM risk.

## 6. Changes in Blood Lipids and Lipoproteins

Nut consumption can favourably alter blood lipids and lipoproteins. Mediterranean diets enriched with 30 g/day of nuts after 1 year of follow up in subjects with a high CVD risk showed an increase in cholesterol efflux capacity and an increase in the percentage of large HDL-C particles [153].

Meta-analyses of nut intervention studies showed a favourable alteration in lipid profiles and apolipoproteins [9,154,155,156]. A meta-analysis of 61 interventions with the study period ranging from 3 to 26 weeks [9] showed that nut consumption reduced TC (−4.7 mg/dL), LDL-C (−4.8 mg/dL), apolipoprotein B (apo B) (−3.7 mg/dL), and triglyceride (TG; −2.2 mg/dL) regardless of type of nut or background diets. A favourable effect on Apo B was stronger in individuals with T2DM. An association between tree nut consumption and a reduction in TC and LDL-C was seen at ≥60 g/day of nuts. The LDL-C lowering effect of 100 g/day of nuts was up to 35 mg/dL (0.9 mmol/L) [9]. Coronary deaths may be reduced by 20% (95% CI: 0.76, 0.85) per 1.0 mmol/L reduction in LDL-C [157]. Moreover, a separate meta-analysis of only almonds [154] or only walnut [156] intervention studies showed a significant reduction in TC, LDL-C and TG and no alteration in HDL-C [154] and reduction in TC, LDL-C and no alteration in HDL-C and TG compared with the diet [156]. Incorporation of 50–100 g/day nuts providing 35% of energy to a low-fat diet five or more times per week can lower TC and LDL-C for the prevention of CVD [158,159,160].

In a randomised, cross-over, controlled study, which was not included in the meta-analyses [9,154,155,156], comparing three 4-week iso-energetic dietary regimes in subjects with an increased risk of CVD, a diet providing 20% of energy from pistachios (63–126 g/day of pistachios; 34% total fat and 8% SFA) significantly reduced small, dense LDL-C levels (sdLDL, associated with more CVD events than total LDL-C) and the triacylglycerol:HDL-C ratio compared with the low-fat control diet (control; 25% total fat and 8% SFA), as well as improved ATP-binding cassette transporter A1 (ABCA1)-mediated serum cholesterol efflux capacity but only in participants with low hs-CRP status at baseline (<10 mg/L), compared with a diet providing 10% of energy from pistachios (32–63 g/day of pistachio; 30% total fat and 8% SFA) [74]. 

Favourable effects of nut consumption on lipid profiles could in part result from unsaturated fat or bioactive compounds including plant sterols and dietary fibre [158,161]. Meta-analyses of prospective cohort studies showed that PUFAs in nuts (especially in walnuts) and ALA (*n*-3 PUFA; 18:3*n*-3) were inversely associated with the risk of CVD [162,163].

### 6.1. Unsaturated Fat

Unsaturated fat in nuts may alter the composition of very low density lipoprotein (VLDL) [164] and the activities of lipoprotein lipase and hepatic lipase [165,166] and decrease apo C-III in HDL.

Park et al. demonstrated that PUFAs decreased the expression of cholesterol transporter NPC1Ll in Caco-2 cells (human colon adenocarcinoma cell line) and HepG2 cells (human liver carcinoma cell line) compared with SFAs and phytosterols [167]. Harini et al. [168] proposed several potential mechanisms for PUFA to lower lipids. The expression of several genes involved in lipid metabolism can be mediated by PUFAs via nuclear receptors including the nuclear receptors (PPAR), liver X receptor (LXR), and hepatocyte nuclear factor-(HNF)-4α], via NFκB and the transcription factors sterol-regulatory element binding protein (SREBP) [168].

### 6.2. The Lysine to Arginine Ratio

One potential mechanism of the hypolipidemic effects of nuts may be the low content of lysine and the relatively high content of arginine. In animals, a diet with a low Lys: Arg ratio promoted 7-α hydroxylase activity resulting in elevated bile acid production and excretion of neutral and acidic steroids decreasing the hepatic cholesterol pool size. This can result in a decrease in LDL-C [169,170].

### 6.3. Phytosterols

A meta-analysis of RCTs showed that a daily intake of approximately 3 g phytosterols lowered LDL-C concentrations by 12% [171]. Possible molecular mechanisms have been proposed but they are still controversial [172]. Phytosterols are plant-specific phytochemicals and their chemical structure is similar to that of cholesterol. Phytosterols have been known to compete against dietary cholesterol and biliary cholesterol incorporation into mixed micelles in the intestinal lumen [172,173,174].

Intestinal transport proteins and receptors have also been proposed as a mechanism underlying the cholesterol-lowering effect of phytosterols [172]. The NPC1L1 (a target of ezetimibe which lowers cholesterol uptake) is a protein expressed in jejunal enterocytes and localized to the brush border membrane and which is required for intestinal cholesterol absorption and phytosterol absorption [175,176].

On the other hand, the heterodimer of ATP-binding cassette (ABC) transporters, G5 (ABCG5) and G8 (ABCG8) inhibit the absorption of cholesterol and phytosterols from the diet by driving the efflux of cholesterol and phytosterols from enterocytes back into the intestinal lumen, and by facilitating the release of cholesterol and phytosterols from hepatocytes into bile [177].

Intestinal acyl CoA: cholesterol acyltransferase (ACAT2) converts cholesterol to cholesteryl ester which is packed into chylomicrons by microsomal triglyceride protein (MTP—a lipid transfer protein) for secretion into chyle [178]. In hamsters fed a high cholesterol diet, β-sitosterol and stigmasterol down-regulated mRNA levels of intestinal ACAT2 and MTP [179]. Brauner et al. [180] demonstrated that phytosterols have lower uptake and lower esterification by ACAT2 in the intestine than cholesterol. In Caco-2 enterocytes, phytosterols decreased cholesterol absorption by suppressing 27-hydroxycholesterol generation by CYP27, liver X receptor α (LXRα) and the basolateral sterol exporter ABCA1 expression, and delivering sterol back into the gut lumen by ABCG5/G8 [180]. 

3-hydroxy-3-methylglutary-CoA (HMG-CoA) reductase is the rate-limiting enzyme in cholesterol synthesis. HMG-CoA reductase gene expression was reduced in CaCo-2 cells incubated with beta-sitosterol [181]. An in vitro study showed that stigmasterol, campesterol and β-sitosterol decreased secretion of apo B48 in Caco2 human intestinal cells by 15%, 16% and 19% respectively. Moreover, those three phytosterols led to a 30% reduction in VLDL levels as measured by secretion of apo B100 in HepG2 human liver cells [182].

In a placebo-controlled, crossover feeding trial, phytosterol intakes at moderate (458 mg/day) and high (2059 mg/day) doses significantly increased total faecal cholesterol excretion and biliary cholesterol excretion and decreased intestinal cholesterol absorption [183].

### 6.4. Fiber

Meta-analyses of prospective cohort studies showed that total dietary fibre consumption (average of 7 g/day) was inversely associated with the risk of CVD [184,185]. Insoluble fiber in nuts increases satiety and faecal bulk. Soluble fiber (viscous fiber) decreases gastric emptying and impairs diffusion across the unstirred water layer in the small intestine and increases bile acid excretion [186]. These favourable effects of fiber may partly contribute to the cholesterol-lowering effects of nuts.

## 7. Antioxidant Activity and Decreased Oxidative Stress

Nuts have very different antioxidant capacities varying from 1.2 to 120 mg of Trolox equivalents per 100 g fresh weight [19]. In a systematic review of in vitro, in vivo and human intervention studies, most nuts showed favourable effects on oxidation while walnuts showed inconsistent antioxidant effects [187]. The PREDIMED study showed that adherence to the Mediterranean diets enriched with either an average of 50 mL/day of virgin olive oil or 30 g/day of nuts for 5 years in subjects with metabolic syndrome decreased xanthine oxidase (an enzymatic source of reactive oxygen species (ROS)) activity and increased plasma SOD and catalase activities compared with a low-fat diet [188].

MUFAs which are abundant in nuts resist oxidation [189,190] while PUFAs from walnuts which are potentially very oxidisable due to their double bonds [191] appear not to influence LDL oxidation compared with a walnut-free, lower-PUFA diet [68,192,193,194], indicating that other bioactive compounds in nuts such as polyphenols [195,196,197,198], tocopherols [199], phytosterols (β-sitosterol) [200,201] and selenium [202,203] could be possible candidates for the antioxidative effects, possibly in a synergistic manner [204]. Human interventions evaluating the effect of nut consumption on oxidative stress are shown in Table 4. Thirteen [24,71,77,121,203,205,206,207,208,209,210,211,212] of 14 clinical studies in Table 4 showed a protective effect on oxidative stress. Only one study [213] showed no effect on oxidative stress.

Walnuts have the highest content of polyphenols in both raw and roasted nuts. Walnut polyphenols have the highest antioxidant efficacy compared with other nuts. Walnuts, almonds, Brazil, cashews, hazelnuts, macadamias, peanuts, pecans, pistachios and peanut butter have greater antioxidant power compared with α-tocopherol ranging from 15 times for raw walnuts to 1.9 times for roasted cashews [14].

In in vitro studies, 14 phenolic compounds obtained from walnuts showed SOD-like activity and 1,1-diphenyl-2-picrylhydrazyl (DPPH) radical scavenging activity [195].

Walnut extracts consisting of ellagic acid monomers, polymeric ellagitannins and other phenolics, principally nonflavonoid compounds inhibited Cu^2+^-induced LDL oxidation by 84% while ellagic acid alone at the same level inhibited Cu^2+^-induced LDL oxidation by 14% [196]. Approximately 120 phenolic compounds have been identified in walnuts [214]. Almond polyphenols and α-tocopherol synergistically inhibited Cu^2+^-induced LDL oxidation [198,204]. A decrease in Cu^2+^-induced LDL oxidation was also observed in clinical studies following 4 weeks of almond consumption (60 g/day) in men with mild hyperlipidemia [215] and in Chinese patients with T2DM [216] but not in normocholesterolemic healthy subjects who consumed 66 g/day of almonds for 6 weeks [65].

Walnuts and cashews (63–108 g/day for each nut) significantly improved antioxidant status with a reduction in oxidized glutathione (GSSG) and an increase in oxygen radical absorbance capacity (ORAC) levels but only in comparison with baseline and not the control diet [206]. An acute study examined postprandial oxidative stress compared a walnut meal containing 90 g walnuts and a isocaloric control meal (50% carbohydrate; 20% protein; 30% fat) in healthy young subjects. The walnut meal reduced oxidative stress by significantly increasing iAUC for hydrophilic and lipophilic ORAC and decreasing iAUC for MDA over the course of a 5-h test. There was decreased oxidized LDL at 2 h after a walnut meat. The walnut meal also significantly elevated plasma levels of epicatechin gallate (ECG), epigallocatechin gallate (EGCG) and gallocatechin gallate (GCG) 1 h after a walnut meal. A significant amount of urolithin-A in urine samples was detected after the walnut meal. A diet effect for plasma γ-tocopherol but not for α-tocopherol was observed in the mixed linear models [211]. 

An acute study showed that the glucose and insulin-lowering effects of almonds were also related to a lower risk of oxidative damage to proteins [32]. An almond meal improved postprandial protein damage by increasing serum protein thiol (-SH) levels compared with control meals (bread, potato and rice meals containing similar amounts of carbohydrate, protein and fat) when data from three control meals were combined. There were negative correlations between postprandial changes in protein thiols and 2-h glucose iAUC or 2-h insulin iAUC or glucose peak height or insulin peak height, indicating that blood glucose levels increase as damage to protein thiols increases [32].

Four-week pecan [212] and 3-week pistachios [71] consumption in healthy individuals also showed decreased MDA concentrations. Pistachio diets (32–63 or 63–126 g/day) consumed by hypercholesterolemic subjects for 4 weeks significantly reduced serum oxidized-LDL levels compared with a low-fat control diet and elevated plasma lutein, α-carotene, and β-carotene levels compared with baseline [210]. Daily supplementation of two Brazil nuts added to a normal diet for 12 weeks increased plasma selenium concentrations and GPx activities compared with the normal diet [203]. In addition, the consumption of pistachios (65–75 g/day) for 3 weeks and hazelnuts (1 g/kg body weight/day) for 30 days in healthy subjects [71,77] and macadamias (40–90 g/day) for 4 weeks in hypercholesterolemic subjects [207] enhanced oxidative status but these studies had the limitation that the amount of SFA in the nut diet was lower than that in a control diet. Nuts also reduce oxidative DNA damage [121,205,208].

However, whether the antioxidant effects of nuts play a role in their apparent effects in CVD prevention is not clear and more clinical trials of nuts with hard endpoints are required.

## 8. Anti-Inflammatory Actions

Elevated levels of pro-inflammatory markers (e.g., hs-CRP, IL-6 and TNF-α) are strong predictors of the development of T2DM [217,218] and CVD [219]. In the Nurses’ Health Study (NHS) and the Health Professionals Follow-Up Study (HPFS) of US men and women with no diabetes, nut consumption (≥5 times per week, 28 g per serving) was associated with lower inflammatory markers of hs-CRP (relative concentrations 0.80 (95% CI: 0.69, 0.90); *p* = 0.0003) and IL-6 (0.86 (95% CI: 0.77, 0.97); *p* = 0.006) after adjusting for demographic, medical, dietary, and lifestyle variables, compared with no or almost no nut consumption [220]. A sub-study of the PREDIMED study (two Mediterranean diets enriched with either 50 mL/day of virgin olive oil or 30 g/day of nuts and a control lower fat Mediterranean diet) investigated methylation changes in inflammation-related genes in peripheral blood cells between baseline and 5 years in 36 subjects at high cardiovascular risks. This study showed a correlation between methylation changes of eight genes (EEF2, COL18A1, IL4I1, LEPR, PLAGL1, IFRD1, MAPKAPK2, and PPARGC1B) related to inflammation and adherence to a Mediterranean diet. A positive correlation between levels of EEF2 methylation and TNF-α and hs-CRP was observed, suggesting that Mediterranean diets might have anti-inflammatory actions possibly via epigenetic mechanisms [221]. Human interventions evaluating the effect of nut consumption on inflammation are shown in Table 5. Most clinical studies in Table 5 showed beneficial effects on inflammatory markers. Only one pistachio study showed no change in inflammatory markers [22]. 

In a pre–post intervention study [226], a diet supplemented with raw almond (20% of total energy substituted for fat and carbohydrate) and physical activity (45 min of walking at least 5 days a week) for 24 weeks in subjects with T2DM showed a significant decrease in hs-CRP (*p* < 0.01), along with a reduction in waist circumference, waist-to-height ratio, serum TC, TG, LDL-C, and HbA1c compared with a baseline 3-week almond-free diet and same level of physical activity [226].

Zhao et al. [222,223] conducted a randomized, controlled cross-over study in 23 hypercholesterolemic subjects who were randomly assigned to one of three diets for 6 weeks each: an average American diet ((AAD)—13% energy from SFA, 13% energy from MUFAs and 8.7% energy from PUFAs (7.7% LA; 0.8% ALA)); two diets high in PUFAs—a LA diet and ALA diet. The LA diet consisted of 16.4% energy from PUFAs (12.6% LA; 3.6% ALA). The ALA diet consisted of 17% energy from PUFAs (10.5% LA; 6.5% ALA). Walnuts and walnut oil were used for half of total fat in the 2 high-PUFA diets. The ALA diet reduced hs-CRP (*p* < 0.01) while the LA diet had a trend to decrease hs-CRP (*p* = 0.08) compared with the AAD [222]. The levels of IL-6, IL-1β and TNF-α cultured in peripheral blood mononuclear cells (PBMCs) was significantly lower on the ALA diet than on the LA diet (*p* < 0.05) [223]. In addition, the ALA diet decreased VCAM-1 by 16% while the LA diet decreased it by only 3% (*p* < 0.01). This study indicates that ALA and EPA derived from ALA exert a key role in the anti-inflammatory responses after walnut consumption [222] but a large proportion of dietary ALA is oxidized after digestion and only a small proportion is converted to EPA/DHA [227].

Other bioactive compounds in nuts may influence anti-inflammatory activity. Ellagic acid, a major polyphenolic compound in especially walnuts, has shown anti-inflammatory activity [228,229]. Nuts are a good source of magnesium which is inversely associated with the risk of CVD [230,231] and hs-CRP [232,233], and E-selectin levels [233].

Hernandez-Alonso et al. [225] showed an anti-inflammatory effect of pistachio consumption (pistachios are higher in β-carotene, γ-tocopherol and lutein than other nuts) compared with a control diet with olive oil to compensate for the energy from pistachios) [225]. Another pistachio diet (57 g/day) for 4 months significantly decreased IL-6 mRNA (*p* = 0.004) and resistin gene expression (*p* = 0.04) in lymphocytes compared with the control diet [225]. Elevated serum resistin levels are associated with insulin resistance, T2DM, and CVD [234,235,236].

Luo et al. [237] demonstrated the mechanism of resistin in increased glucose production and decreased insulin action in human liver HepG2 cells. Resistin led to insulin resistance through both 5′ adenosine monophosphate-activated protein kinase (AMPK)-dependent and AMPK-independent pathways in hepatocytes: resistin upregulated cytokine signalling 3 (SOCS-3) expression, and downregulated insulin receptor substrate 2 (IRS-2) and Akt phosphorylation via an AMPK-independent mechanism. In addition, resistin downregulated AMPK leading to upregulated mRNA expression of gluconeogenic enzymes, G6Pase and PEPCK, and downregulated glucose transporter 2 (GLUT2) mRNA and glycogen synthesis [237].

## 9. Endothelial Function

Oxidative stress (increased F2-isoprostanes and oxidized LDL), inflammation (hs-CRP) and endothelial dysfunction (e.g., increased E-selectin and intercellular cell-adhesion molecule-1 (ICAM-1)) are positively associated with the risk of T2DM [238,239].

Endothelial dysfunction which is characterized by decreased nitric oxide (NO) bioavailability (either decreased NO synthesis or destruction of NO by reactive oxygen species such as superoxide) [240], is associated with obesity and insulin resistance and is involved in initiation and promotion of atherosclerosis [241] and is related to the risk of CVD [242,243].

Insulin can induce both vasodilation and vasoconstriction in the endothelium. It has been postulated that insulin binding to the insulin receptor (IR) activates IR substrate (IRS) proteins leading to activation of phosphoinositide 3-kinase (PI3K)/protein kinase B (PKB)/endothelial NO synthase (eNOS) at the Ser1177-P pathway [244,245,246,247,248]. Activated eNOS synthesizes NO and l-citrulline from l-arginine and molecular oxygen. NO then stimulates cyclic GMP to induce smooth muscle relaxation.

The vasoconstrictive actions of insulin are mediated by endothelin-1 (ET1). ET-1 is a potent endothelial activator which participates in plaque development and smooth muscle proliferation via the mitogen-activated protein kinase (MAPK)/ERK activation and cyclin D1 pathways [249]) through MAPK/ERK, which antagonizes insulin-induced, PI3K-dependent vasodilatation in skeletal muscle arterioles [250]. NO donors can inhibit cytokine-induced endothelial cell activation and monocyte adhesion partly by inhibiting NF-κB [251]. Ellagic acid (50 μmol/L) significantly blocked the expression of VCAM-1 and E-selectin by inhibiting IL-1 β-induced activation of NF-kB p65 and p50, leading to reduced monocyte adhesion in HUVEC [228].

Elevated inflammatory cytokines promote endothelial dysfunction [241]. The inflammatory cytokine TNF-α has been shown to decrease NO bioavailability in the vascular endothelium [252]. TNF-α is also known to increase the expression of cell adhesion molecules such as VCAM-1, ICAM-1 and E-selectin [253]. TNF-α-induced expressions of VCAM-1 and ICAM-1 were significantly inhibited in human aorta endothelial cells (HAEC) by walnut extract (10–200 μg/mL) or ellagic acid (0.1 and 1 μM) [229]. 

Unsaturated fatty acids, especially oleic acid in nuts, appear to prevent atherosclerosis by modulating gene expression of endothelial leukocyte adhesion molecules. In HUVECs, oleic acid suppressed the expression of VCAM-1 stimulated with cytokines (e.g., TNF-α, IL-1α, IL-1β and IL-4) or lipopolysaccharide (LPS) or protein-kinase C activator (PMA) and oleic acid also inhibited LPS-stimulated NF-kB activation. Moreover, oleic acid decreased SFAs (palmitic and stearic acids) and increased oleic acid with no change PUFAs in the cell membrane phospholipids which influences prostaglandin production [254]. 

Adiponectin activates AMPK and PKB signalling resulting in NO generation in endothelial cells [255,256], and suppresses the expression of VCAM-1, ICAM-1 and E-selectin [257]. Walnut consumption (48 g/day) for 4 days increased circulating apo AI and total adiponectin levels in obese subjects with the metabolic syndrome, indicating that even short-term walnut consumption benefits the lipid profile and adiponectin levels which may insulin resistance and CVD [258].

Nut consumption may favourably affect endothelial function. Improved endothelial function was observed after consumption of walnuts in overweight subjects with visceral obesity [259], patients with hypercholesterolemia [192,260], and patients with T2DM [261], as well as after consumption of pistachios in subjects with mild dyslipidemia [262].

In a randomized crossover study of hypercholesterolemic subjects comparing a cholesterol-lowering Mediterranean diet and a walnut diet containing similar energy and fat substituting walnuts for 32% of the energy from MUFA in the Mediterranean diet, walnuts significantly improved endothelium-dependent vasodilation and decreased VCAM-1, TC and LDL-C. Decreased cholesterol correlated with increases in dietary α-linolenic acid and LDL γ-tocopherol levels. Changes in endothelium-dependent vasodilation was inversely correlated with those in the TC/HDL-C ratios suggesting the favourable effect of walnut on endothelial function could be explained in part by improved lipid profiles [192].

A whole-walnut diet (whole walnuts: 61–150 g/kg diet) significantly decreased aortic ET-1 mRNA levels (by up to 75%) and the aortic cholesterol ester compared with the lowest α-tocopherol diet groups (α-tocopherol: 8.1–81 mg/kg diet) in hamsters fed a high-fat atherogenic diet [263].

Other nut bioactive such as l-arginine [264] and magnesium [265,266] are possible candidates to account for improved endothelial function [254]. 

In a cross-sectional study, subjects who consumed more than 7.5 g/day of arginine had a 30% lower likelihood of having an hs-CRP above 3.0 mg/L compared with those who consumed 2.5–5.0 g/day of arginine [267]. Intervention studies [268,269] also showed that dietary l-arginine improved endothelial function and decreased monocyte adhesion to endothelial cells in young men with coronary artery disease [269] and decreased P-selectin, ICAM-1, E-selectin, IL-1 and IL-6 levels in subjects with intractable angina pectoris [268]. Similarly, daily supplementation of 10 g of arginine for 4 weeks improved endothelial function and decreased LDL-C oxidation in subjects with stable coronary artery disease without significant changes in inflammatory markers [270]. 

## 10. Blood Pressure 

A meta-analysis of RCTs [271] showed that nut consumption reduced systolic blood pressure (SBP) by 1.3 mm Hg (*p* = 0.02) and diastolic blood pressure (DBP) in subjects without T2DM. Only pistachios and mixed nuts showed a significant effect on BP [271].

Mediterranean diets enriched with either 350 mL/week of extra virgin olive oil or 30 g/day of nuts lowered BP after a 4-year follow-up period [272] and 24-h ambulatory BP after a 1-year follow-up period [273]. Polyphenol consumption may have been responsible for the hypotensive effects [274]. The reduction in BP in 200 participants was positively associated with an increase in total polyphenol excretion and plasma NO markers and a positive correlation between urinary total polyphenol excretion and plasma NO was observed. These findings suggest that polyphenols could also exert a protective effect on endothelial function [274].

## 11. Conclusions

In conclusion, frequent nut consumption could play a role in reducing the risk of T2DM and CVD through improvement in glucose and lipid metabolism, weight maintenance and improved endothelial function. The protective effect of nuts could be explained by their distinctive nutrient profile and non-nutrient bioactive compounds. However, the specific mechanisms underlying these effects are not fully understood. Further targeted research should be undertaken to clarify the biological mechanisms.

## Figures and Tables

**Table 1:** Nutrient content of raw nuts per 100 g *.

	Almond	Walnut	Hazelnut	Macadamia	Pecan	Pistachio	Brazil Nut	Cashew Nut	Peanut	Pine Nut	Chest Nut
Energy (kcal)	579	654	628	718	691	560	659	553	567	673	213
Carbohydrate (g)	21.55	13.71	16.7	13.82	13.86	27.17	11.74	30.19	16	13.08	45.54
Protein (g)	21.15	15.23	14.95	7.91	9.17	20.16	14.32	18.22	26	13.69	2.42
Lysine (g)	0.568	0.424	0.42	0.018	0.287	1.138	0.49	0.928	0.926	0.54	0.143
Arginine (g)	2.465	2.278	2.211	1.402	1.177	2.134	2.14	2.123	3.085	2.413	0.173
Total fat (g)	49.93	65.21	60.75	75.77	71.97	45.32	67.1	43.85	49	68.37	2.26
Saturated fat (g)	3.8	6.126	4.464	12.061	6.18	5.907	16.134	7.783	7	4.89	0.425
MUFA (g)	31.55	8.933	45.652	58.877	40.801	23.257	23.879	23.797	24	18.76	0.78
PUFA (g)	12.329	47.174	7.92	1.502	21.614	14.38	24.399	7.845	16	34.07	0.894
Total Fiber (g)	12.5	6.7	9.7	8.6	9.6	10.6	7.5	3	8.5	3.7	8.1
Folate (μg)	44	98	113	11	22	51	22	25	240	34	62
Calcium (mg)	269	98	114	85	70	105	160	37	92	16	27
Magnesium (mg)	270	158	163	130	121	121	376	292	168	251	32
Sodium (mg)	1	2	0	5	0	1	3	12	18	2	3
Potassium (mg)	733	441	680	368	410	1025	659	660	705	597	518
Copper (mg)	1.031	1586	1725	0.756	1.2	1.3	1.743	2195	1.144	1.324	0.447
Iron (mg)	3.71	2.91	4.7	3.69	2.53	3.92	2.43	6.68	4.58	5.53	1.01
Zinc (mg)	3.12	3.09	2.45	1.3	4.53	2.2	4.06	5.78	3.27	6.45	0.52
Selenium (μg)	4.1	4.9	2.4	3.6	3.8	7	1917	19.9	7.2	0.7	NA
α-tocopherol (mg)	25.63	0.7	15.03	0.54	1.4	2.86	5.65	0	8.33	9.33	NA
β-tocopherol (mg)	0.23	0.15	0.33	0	0.39	0	0.01	0.03	NA	0	NA
γ-tocopherol (mg)	0.07	20.83	0	0	24.44	20.41	9.56	5.31	NA	11.15	NA
δ-tocopherol (mg)	0.07	1.89	0	0	0.47	0.8	0.63	0.36	NA	0	NA
Total phytosterol (mg)	~198	~110.2	~122	~116	~158.7	~214	~123.8	~151	NA	236.1	22
Stigmasterol	4	0	1	0	3	5	6	0		0	-
Campesterol	5	5	7	8	6	10	2	9		20	-
B-sitosterol	130	87	102	108	117	198	64	113		132	-
δ 5-avenasterol	21	7.3	2.6	-	14.3	-	19.7 (δ avenasterol + B sitostanol)	14		40.1	-
B-sitostanol	4	-	3.9	-	-	-		-		5.9	-
Campestanol	2	2.3	3	-	2.8	-	-	2		3.9	-
Others	32	8.6	2.5	-	15.6	-	31.8	13		34.2	-
Total polyphenol (mg) ^#^	287	1576	687	126	1284	867	224	232.9	NA	NA	NA
Total polyphenol (mg) ^$^	212.9 ± 12.3	1580.5 ± 58	314.8 ± 47.3	497.8 ± 52.6	1463.9 ± 32.3	571.8 ± 12.5	169.2 ± 14.6	316.4 ± 7.0	645.9 ± 47	152.9 ± 14.1	NA
Flavonoids (mg) ^$^	93.5 ± 10.8	744.8 ± 93.3	113.7 ± 30.2	137.9 ± 9.9	704.7 ± 29.5	143.3 ± 18.7	107.8 ± 6.0	63.7 ± 2.1	189.8 ± 13.1	45.0 ± 5.4	NA
Ellagitannins (mg) ^‡^	ND	823 ± 59	ND	ND	301 ± 7	ND	ND	ND	ND	NA	149 ± 3
Proanthocyanidins (mg) ^#^	176	60	491	NA	477	226	NA	2	NA	NA	0
Carotenoids (μg)	2	NA	106	NA	55	332	0	NA	NA	NA	NA
Lutein + zeaxanthin (μg)	1	9	92	0	17	2903	0	22	0	9	NA

* Source: United States Department of Agriculture Nutrient Database for Standard Reference; Nutrient data for 12,061 (nuts, almonds), 12,155 (nuts, walnuts, English), 12,120 (nuts, hazelnuts or filberts), 12,131 (nuts, macadamia nuts, raw), 12,142 (nuts, pecans), 12,151 (nuts, pistachio nuts, raw), 12,078 (nuts, brazilnuts, dried, unblanched), 12,087 (nuts, cashew nuts, raw), 16,087 (peanuts, all types, raw), 12,147 (nuts, pine nuts, dried), 12,097 (nuts, chestnuts, European, raw, unpeeled) [16]. ^#^ Data were obtained from [17], ^$^ data were obtained from [18] and data ^‡^ were obtained from [19]; NA: not available; ND: not detected; MUFA, monounsaturated fatty acids; PUFA, polyunsaturated fatty acids.

**Table 2:** Summary of clinical trials examining the effect of nut consumption on glycemic control.

Description	Reference	Subjects	Study Design/Period	Nut Type	Diet Intervention	Results
Meta-analysis of 12 RCTs for glycemic control	Viguiliouk et al., 2014 [20]	Subjects with T2DM	RCTs/≥3-week follow-up period	Almonds, pistachios, walnuts, pecans, hazelnuts, peanuts, cashews, macadamias	Diet supplemented with tree nuts vs. isocaloric diet without tree nuts	↓ HbA1c (*p* = 0.0003), ↓ fasting glucose,
						↔ fasting insulin, ↔ HOMA-IR after consumption of tree nuts at a median dose of 56 g/day
Chronic studies that were not included in meta-analysis of Viguiliouk et al., 2014 [20]	Scott et al., 2003 [25]	17 subjects with IGT (*n* = 10) or T2DM (*n* = 7)	Parallel, randomised/42 weeks	Almonds	Almond-enriched diet (High Protein-High MUFA diet; 25% of energy from protein, 40% fat, and 22% MUFAs) vs. AHA diet (15% of energy from protein, 30% fat, and 15% MUFAs)	↑ glucose control
						↔ TG, ↔ LDL-C, ↔ fasting glucose
	Wien et al., 2003 [26]	65 overweight and obese subjects	Randomised prospective/24 weeks	Almonds	Formula-based low-calorie diet enriched with 84 g/day of almonds vs. complex carbohydrates matching calories and protein	↓ 62% body weight/BMI,
						↓ 50% waist circumference,
						↓ 56% greater reduction in fat mass compared with the complex carbohydrate diet
						↓ glucose, ↓ insulin, ↓ HOMA-IR, ↓ TC, ↓ TG, ↓ LDL-C, ↓ LDL-C/HDL-C in both diets
	Sari et al., 2010 [24]	32 healthy young men	Dietary intervention/8 weeks	Pistachios	Subjects consumed a Mediterranean diet for 4 weeks and in the next 4 weeks they supplemented the Mediterranean diet with pistachios for 4 weeks replacing 32% of the energy obtained from MUFAs in the control diet (20% of energy)	↓ glucose levels (−8.8 ± 8.5% *p* < 0.001)
						↓ TC (*p* < 0.001), ↓ LDL-C (*p* < 0.001), ↓ triacylglycerol, ↔ HDL-C,
						↓ TC-C/HDL-C, ↓ LDL-C/HDL-C (*p* < 0.001),
						↑ endothelium-dependent vasodilation (*p* = 0.002)
						↓ lipid hydroperoxide (*p* < 0.001), ↓ MDA (*p* < 0.001), ↓ IL-6 (*p* < 0.001),
						↑ SOD (*p* < 0.001),
						↔ hs-CRP, ↔ TNF-α
						compared with a Mediterranean diet
	Wien et al., 2010 [36]	65 subjects with prediabetes	Randomized parallel trial/16 weeks	Almonds	ADA diet containing 20% of energy from almonds (60 g/day of pre-packaged raw or dry roasted almonds) vs. almond-free control diet	↓ fasting insulin (*p* = 0.002), ↓ HOMA-IR (*p* = 0.007), ↓ HOMA-B (*p* = 0.001), ↔ fasting glucose, ↓. LDL-C, ↔ BMI,
						↓ weight, ↔ waist circumference
	Parham et al., 2014 [23]	48 subjects with T2DM	Double-blind, randomized, placebo-controlled, crossover/12 weeks	Pistachios	25 g pistachio nuts twice a day as a snack vs. control diet without nuts	↓ HbA1c (−0.4%; *p* ≤ 0.001), ↓ fasting blood glucose (−16 mg/dL; *p* ≤ 0.001),
						↔ BMI, ↔ BP, ↔ HOMA-IR, ↔ hs-CRP
	Sauder et al., 2015 [22]	30 subjects with well-controlled T2DM	Randomized, crossover, controlled/4 weeks	Pistachios	Healthy diet with pistachios contributing 20% of total energy. Low-fat or fat-free snacks (e.g., pretzels) in the control diet were substituted with roasted pistachios providing 20% of daily energy (59 to 128 g/day of pistachios). Half of amount of pistachio was ingested unsalted vs. AHA Therapeutic Lifestyle Changes diet (26.9% total fat, 6.7% SFA, 186 mg/day cholesterol).	↓ fructosamine, ↓ TC, ↓ ratio of TC to HDL-C, ↓ TG (*p* = 0.003), ↔ HbA1c,
						↔ fasting glucose, ↔ insulin, ↔ hs-CRP,
						↔ ICAM, ↔ VCAM, ↔ endothelial function
	Zibaeenezhad et al., 2016 [21]	100 subjects with T2DM	Randomized control/3 months	Walnut oil	Walnut oil (15 g/day) group (*n* = 50) vs. a control group without any interventions (*n* = 50)	↓ HbA1c by 8 ± 22% (*p* = 0.005) and
						↓ fasting glucose by 8 ± 17% (*p* = 0.001) within the walnut group.
						But, ↔ HbA1c, ↔ fasting glucose compared with the control.
						↔ body weight, BMI, BP in the two groups
Acute studies that were not included in meta-analysis	Johnston et al., 2005 [30]	11 healthy subjects	Randomised crossover	Peanuts	6 meals (bagel and juice meal, bagel and juice meal + vinegar, bagel and juice meal + peanut, chicken and rice meal, chicken and rice meal + vinegar, and chicken and rice meal + peanut) were compared	↓ glucose response in the 60-min (*p* < 0.05) following the ingestion of vinegar or peanuts with bagel and juice meal
	Jenkins et al., 2006 [32]	15 healthy subjects	Meals consumed in random order on separate days, 4-h postprandial test	Almonds	3 test meals; almonds (60 g) + bread (97 g), parboiled rice meal (68 g cheese + 14 g butter + 60 g parboiled rice) and mashed potato meal (62 g cheese + 16 g butter + 68 g mashed potatoes) vs. 2 bread control meals	↓ postprandial glucose and insulin (*p* < 0.001) responses
						↑ protein thiol
						↔ total antioxidant capacity compared with other test meals
	Josse et al., 2007 [33]	9 healthy subjects	Meals consumed in random order on separate days, 2-h postprandial test	Almonds	White bread + 0 g almond, white bread + 30 g almond, white bread + 60 g almond, white bread + 90 g almond,	↓ meal glycemic index in a dose-dependent manner (90 g almond + white bread > 60 g almond + white bread > 30 g almond + white bread; *p* = 0.001)
					Each meal contained 50 g CHO from white bread	
	Kendall et al., 2011 [35]	14 normoglycemic subjects and 10 subjects with T2DM	Meals consumed in random order on separate days, 2-h postprandial test	Mixed nuts	Mixed nuts (30, 60 and 90 g) were consumed with white bread	↓ postprandial glycemic responses
					Each meal contained 50 g CHO from white bread	
	Kendall et al., 2011 [27]	10 healthy adults	Two acute tests; each test consisting of 2-h postprandial test	Pistachios	<Study 1> 8 test meals: white bread (50 g CHO), 28 g pistachio, 56 g pistachio, 84 g pistachio, white bread + 28 g pistachios, white bread + 56 g pistachios and white bread + 84 g pistachios, aiming to assess the dose-response effect of 28, 56 and 84 g pistachios ingested alone or with white bread. <Study 2> 7 test meals: white bread (50 g CHO), parboiled rice, pasta and instant mashed potatoes and parboiled rice + 56 g pistachios, pasta + 56 g pistachios instant mashed potatoes + 56 g pistachios, aiming to assess effective 56 g of pistachio on glycemic response with different types of CHO foods	Study 1: relative glycemic responses; for 28 g, 56 g and 84 g pistachios were 5.7%, 3.8% and 9.3% respectively compared to white bread, ↓ relative glycemic responses; in a dose-dependent manner with 89% (*p* = 0.1), 67% (*p* = 0.009) and 52% (*p* < 0.001) for 28 g, 56 g and 84 g pistachios added to white bread respectively Study 2: ↓ relative glycemic responses; after adding 56 g pistachios to rice, pasta and instant mashed potatoes with 59% (*p* = 0.02), 56% (*p* = 0.025) and 87% (*p* = 0.06) respectively, compared with 73%, 95%, 109%
	Mori et al., 2011 [31]	14 subjects with IGT	Randomized, 5-arm, crossover	Almonds	Whole almonds into breakfast vs. almond butter into breakfast vs. defatted almond flour into breakfast vs. almond oil into breakfast vs. no almonds into breakfast	↓ postprandial glycemia and ↑ satiety following whole almonds ↓ the second-meal NEFA response following whole almonds or almond oil ↔ GLP-1 between meals
					All breakfast meals were matched for 75 g carbohydrate	
	Reis et al., 2013 [29]	15 obese women with a high T2DM risk	Randomised cross-over	Peanuts	Consumption of a 75 g CHO-matched breakfast containing 42.5 g of whole peanuts without skins or peanut butter or no peanuts to meal and then consumption of standard lunch, aiming to assess first- and second meal responses	↓ NEFA iAUC _(0–240 min)_, ↓ glucose iAUC _(240–490 min)_, ↑ insulin _(120–370 min)_ ↑ gut satiety hormones (PYY, GLP-1 and CCK), ↓ desire to eat following peanut butter breakfast compared with no peanut breakfast
	Kendall et al., 2014 [28]	20 subjects with metabolic syndrome	Randomized crossover	Pistachios	5 test meals:	↓ glucose response ↑ GLP-1
					- 3 meals containing 50 g available CHO: white bread, white bread, butter and cheese, and white bread and pistachios	
					- 2 meals containing12 g available CHO: white bread, and pistachios	
	Crouch et al., 2016 [34]	20 subjects with prediabetes or isolated 1-h postprandial hyperglycemia		Almonds	one-half ounce (14.2 g) dry-roasted almond preload vs. without the almond preload.	↓ glucose 1 h after a 75-g glucose challenge (*p* < 0.001).

ADA, American Diabetes Association; AHA, American Heart Association; BP, blood pressure; BMI, body mass index; CCK, cholecystokinin; CHO, carbohydrate; GLP-1, glucagon-like-peptide 1; HbA1c, glycosylated haemoglobin; HDL-C, high-density lipoprotein cholesterol; HOMA-IR, homeostasis model assessment of insulin resistance index; HOMA-B, homeostasis model analysis for beta-cell function; hs-CRP, high sensitivity C-reactive protein; iAUC, incremental area under curve; ICAM, intracellular adhesion molecule; LDL-C, low density lipoprotein; IGT, impaired glucose tolerance; IL-6, interleukin-6; MDA, malondialdehyde; MUFA, monounsaturated fatty acids; NEFA, non-esterified fatty acids; PYY, peptide YY; RCT, randomised controlled trial; SFA, saturated fatty acid; SOD, superoxide dismutase; TC, total cholesterol; TG, triglyceride; VCAM, vascular cellular adhesion molecule; ↑, increase; ↓, decrease; ↔, no effect.

**Table 3:** Summary of clinical trials examining the effect of nut consumption on body weight control.

Reference	Subjects	Study Design/Period	Nut Type	Diet Intervention	Results
Spiller et al., 1992 [64]	26 subjects with hypercholesterolemia	Pre-post supplemental, dietary advice/9 weeks	Almonds	Raw almonds (100 g/day; 34 g/day MUFA, 12 g/day PUFA 6 g/day SFA) to baseline diet	↔ body weight, ↑ 81 kcal/day energy intake,↓ LDL-C, ↓ TC (*p* < 0.001), ↔ HDL-C
Abbey et al., 1994 [53]	16 male health subjects	Pre-post consecutive supplemental, dietary advice/9 weeks	Almonds, Walnuts	In the first 3-week period, addition of raw peanuts (50 g/day), coconut cubes (40 g/day), and a coconut confectionary bar (50 g/day), to Australian diet (reference diet)	↔ body weight, ↔ energy intake ↓ LDL-C, ↓ TC, ↔ HDL-C compared with reference diet
				During the following 3 weeks, 84 g/day almonds added to Australian diet	
				During the final 3-week period, 68 g/day walnuts added to Australian diet	
Colquhoun et al., 1996 [76]	14 subjects with hypercholesterolemia	Randomised crossover, pre-post, dietary advice/4 weeks	Macadamias	Diet enriched with macadamia (40% energy as fat, 20% energy from macadamia nuts) vs. a high-complex-carbohydrate diet	↔ body weight, ↔ energy intake
					↓ LDL-C (*p* < 0.01), ↓ TC (*p* < 0.01) on both diets compared with baseline.
					↔ HDL-C on both diets
Spiller et al., 1998 [63]	45 subjects with hypercholesterolemia	Randomised parallel arm, dietary advice/4 weeks	Almonds	Almond-based diet vs. olive oil-based diet vs. dairy-based diet	↔ body weight, ↔ energy intake
					↓ TC (*p* < 0.001), ↓ LDL-C (*p* < 0.001) compared with control diets
				Three diets were matched for total fat	↓ TC, ↓ LDL-C (*p* < 0.001), ↓ TC:HDL ratio (*p* < 0.001), ↔ HDL-C within the almond group
Chisholm et al., 1998 [67]	21 subjects with hypercholesterolemia	Randomised crossover, dietary advice/4 weeks	Walnuts	20% energy of low-fat diet replaced with 78 g/day walnuts vs. low-fat diet	↔ body weight, ↔ energy intake, ↓ TC, ↓ LDL-C, ↑ HDL-C on both diets compared with baseline.
					↓ apo B on the walnut diet compared with low-fat diet
Durak et al., 1999 [77]	30 healthy subjects	Pre-post supplemental, dietary advice	Hazelnuts	1 g/kg body weight/day of hazelnuts supplemented with a habitual diet vs. baseline	↑ 0.5 kg body weight, ↓ TC (*p* < 0.005), ↓ LDL-C (*p* < 0.0005),
					↑ HDL-C, ↑ TG (*p* < 0.001), ↑ HDL-C/LDL-C,(*p* <,0.0005)
					↓ MDA(*p* < 0.0005), ↑AOP (*p* < 0.0005) after hazelnut supplementation
Edwards et al., 1999 [72]	10 subjects with hypercholesterolemia	Randomised, controlled, crossover, dietary advice/3 weeks	Pistachios	20% energy of habitual diet replaced with pistachios vs. control diet	↔Body weight, ↔ BP, ↔ energy intake, ↓ TC, ↓ LDL-C, ↓ TC/HDL (*p* < 0.01), ↑ HDL-C, ↔ TG
Morgan et al., 2000 [70]	19 subjects with normal lipid levels	Randomised parallel arm, pre-post, supplemental/8 weeks	Pecans	68 g/day pecans + self-selected diet vs. self-selected diet without nuts	↔ body weight, ↑ 71 kJ energy intake
					↓ TC, ↓ HDL-C
Zambone et al., 2000	49 subjects with hypercholesterolemia	Randomised crossover, dietary advice/6 weeks	Walnuts	18% energy of habitual diet replaced with walnuts vs. Mediterranean diet	↔ body weight, ↔ energy intake
					↓ TC (*p* < 0.001), ↓ LDL-C (*p* < 0.00), ↓ lipoprotein (a) compared with the Mediterranean diet,
Curb et al., 2000 [75]	30 healthy subjects	Randomized crossover/30 days	Macadamias	3 dietary periods; American diet high in saturated fat (37% energy from fat) vs. AHA Step 1 diet (30% energy from fat) vs. MUFA diet rich in macadamia (37% energy from fat)	↔ body weight, ↑ energy intake
					↓ TC, ↓ LDL-C after macadamia diet compared with American diet
Almario et al., 2001 [52]	18 subjects (7 men and 16 postmenopausal women) with dyslipidaemia	Pre-post consecutive supplemental, dietary advice/6 weeks	Walnuts	48 g/day walnuts added to a habitual diet vs.	↔ body weight, ↑ energy intake
				48 g/day walnuts added to a low-fat diet vs.	↓ TC after walnut plus a low-fat diet compared with habitual diet or low-fat diet
				habitual diet vs. low-fat diet	↓ LDL-C (*p* < 0.01) after walnut plus a low-fat diet compared with a low-fat diet
McManus et al., 2001 [79]	101 overweight subjects	Randomized controlled/18 months	Peanuts, almonds, cashews, hazelnuts, macadamias, pecans, pistachios, walnuts	Moderate fat low energy diet (fat: 35% of energy; predominantly PUFAs; nut-enriched diet) vs. low fat, low-energy diet (fat: 20% of energy)	↓ 4.1 kg body weight, ↓ 1.6 kg/m^2^ BMI, ↓ 6.9 cm waist circumference in moderate fat group, while ↑ 2.9 kg body weight, ↑ 1.4 kg/m^2^ BMI, ↑ 2.6 cm waist circumference in low fat group
Fraser et al., 2002 [50]	81 male and female subjects	Randomized crossover/6 months	Almonds	42–70 g/day almond supplementation (320 kcal/day) vs. without almond supplementation	↑ 0.40 kg body weight
Jenkins et al., 2002 [62]	27 subjects with hypercholesterolemia	Randomised crossover, dietary advice/4 weeks	Almonds	3 isoenergetic (mean 423 kcal/day) supplement periods each for 4 weeks; - full-dose almonds (73 g/day), - half-dose almonds plus half-dose muffins, - full-dose muffins (control) Supplements provided 22.2% of energy	↔ body weight, ↔ energy intake
					↓ LDL-C (*p* < 0.001), ↓ LDL-C:HDL-C (*p* < 0.001) on both almonds
					↓ oxidised LDL (*p* < 0.001), ↓ lipoprotein (a) on full -dose almonds
					↔ pulmonary nitric oxide between control, half-dose, and full-dose almond diet
Hyson et al., 2002 [65]	22 subjects with normal lipid levels	Randomised crossover, pre-post, supplemental/6 weeks	Almonds	50% of fat energy of habitual diet replaced with almonds (~66 ± 5 g/day), vs. 50% of fat energy of habitual diet replaced with almond oil (~35 ± 2 g/day)	↔ body weight, ↔ energy intake
					↓ TC, ↓ LDL-C, ↓ TG, ↑ HDL compared with baseline
Morgan et al., 2002 [69]	42 subjects with borderline high total cholesterol	Randomised crossover, dietary advice/6 weeks	Walnuts	64 g/day of walnuts added to low-fat, low-cholesterol diet vs. low-fat, low-cholesterol diet	↔ body weight, ↔ energy intake
					↓ TC, ↓ LDL-C, ↓ TG, ↑ HDL-C
					↔ homocysteine ,↓ tPA, ↓PAI-1
Lovejoy et al., 2002 [37]	20 healthy subjects	Pre-post supplemental study, dietary advice/4 weeks	Almonds	100 g/day of almond added to habitual diet vs. baseline	↑ body weight (↑ 0.9 kg; male, ↑ 0.3 kg: female), ↑ ~51 kJ energy intake, ↓ LDL-C (*p* = 0.0004), ↔ insulin sensitivity compared with baseline
Alper et al., 2002 [49]	15 healthy subjects with normal weight	Crossover, 3 treatment phases (free-feeding, addition and substitution)/30 weeks	Peanuts	In the free-feeding phase, 50% of dietary fat energy from peanuts for 8 weeks with no restriction of the background diet. No dietary advice given.	↔ body weight during the substitution phase
				In the addition phase, peanuts added to their habitual diet for 3 weeks with dietary instructions	↑ 1 kg body weight during the free-feeding phase
				In a substitution phase, an equivalent quantity of fat from peanuts substituted for fat in the diet for 8 weeks. Peanuts consumed at average of 89 ± 21 g/day equivalent to 2113 ± 494 kJ/day (505 ± 118 kcal/day) during the 3 treatment phases. 50% dietary fat energy provided by peanuts.	↑ resting energy expenditure by 11% after adjustment for changes in body weight after peanut consumption for 19 weeks (*p* < 0.01)
Wien et al., 2003 [26]	65 overweight and obese subjects	Randomised prospective/24 weeks	Almonds	Formula-based low-calorie diet enriched with 84 g/day of almonds vs. complex carbohydrates matching calories and protein	↓ 62% body weight/BMI,
					↓ 50% waist circumference,
					↓ 56% greater reduction in fat mass compared with the complex carbohydrate diet
					↓ glucose, ↓ insulin, ↓ HOMA-IR,↓ TC, ↓ TG, ↓ LDL-C, ↓ LDL-C/HDL-C in both diets
Pelkman et al., 2004 [80]	53 overweigh and obese subjects	Parallel-arm/10 weeks (6 weeks for weight loss and additional 4-week for weigh maintenance)	Peanuts	Moderate-fat (33% of energy) diet vs. low-fat (18% of energy) diet	↓ 1.2 ± 0.05 kg/week body weight within the moderate-fat group
					↓ 1.09 ± 0.06 kg/week body weight within the low-fat diet groups
					No difference in weight loss between two groups.
					↔ HDL-C, ↓ triacylglycerol,
					non-HDL C/HDL-C within the moderate-fat diet group for 6 weeks
					↑ triacylglycerol, ↓ HDL-C, ↔ non-HDL-C/HDL-C within the low-fat diet group for 6 weeks
Sabate et al., 2005 [66]	90 healthy subjects	Randomized cross-over/6 months	Walnuts	28–56 g/day walnut-supplementation (12% energy intake) vs. control diet without nuts	↑ energy intake (133 kcal), ↑ 0.4 kg body weight
Kocyigit et al., 2006 [71]	44 health subjects	Randomised parallel arm/3 weeks	Pistachios	20% energy of regular diet replaced with pistachios vs. regular diet	↔ body weight, ↔ energy intake
					↓ TC, ↓ MDA, ↓ TC/HDL-C (*p* < 0.001)
					↓ LDL-C/HDL-C (*p* < 0.01), ↑ HDL-C (*p* < 0.001), ↑AOP, ↑AOP/MDA
Hollis et al., 2007 [51]	20 healthy female subjects	Randomised crossover, no dietary advice/10 weeks	Almonds	1440 kJ portion of raw, unsalted almonds added to a habitual diet vs. habitual diet	↔ body weight, ↔ energy expenditure
Wang et al., 2012 [73]	90 subjects with metabolic syndrome	Randomised parallel arm, dietary advice/12 weeks	Pistachios	42 g/day pistachios plus AHA Step 1 diet vs. 70 g/day pistachio plus AHA Step 1 diet vs. AHA step 1 diet	↔ body weight,↔ fasting glucose, ↔ 2-h postprandial glucose among treatments
					↓ 2-h postprandial glucose within the pistachio groups
					↔ TC, ↔ TG (*p* = 0.018), ↔ LDL-C, ↔HDL-C
Holligan et al., 2014 [74]	28 subjects with elevated LDL levels	Randomised, cross-over, controlled-/4 weeks	Pistachios	20% energy of a diet replaced with 63–126 g/day of pistachios vs. 10% energy of a diet replaced with 32–63 g/day of pistachios vs. low-fat control diet	↔ body weight, ↔ energy intake
					↓ small, dense LDL-C levels compared with the low-fat control diet.
					↓ triacylglycerol/HDL-C on 63–126 g/day of pistachio diet compared with the control diet
Razquin et al., 2017 [78]	4242 subjects	Randomised controlled/3 years	Walnuts, Almonds, Hazelnuts	Mediterranean diets enriched with 30 g/day of nuts (15 g/day walnuts, 7.5 g/day hazelnuts, and 7.5 g/day almonds) vs. Mediterranean diets enriched with 50 mL/day of extra virgin olive oil vs. low-fat diet	↓ body weight
					(↓ ~5 kg in the two lowest quintiles of energy density
					↓ ~4 kg the highest quintile of energy density
					↓ ~2 kg in the two middle quintiles of energy density)

AOP, antioxidant potential index; AHA, American Heart Association; BMI, body mass index; BP, blood pressure; HDL-C, high-density lipoprotein cholesterol; LDL-C, low density lipoprotein; MDA, malondialdehyde; MUFA, monounsaturated fatty acids; PAI-1, plasminogen activator inhibitor-1; SFA, saturated fatty acid; TC, total cholesterol; TG, triglyceride; tPA, tissue plasminogen activator; ↑, increase; ↓, decrease; ↔, no effect.

**Table 4:** Summary of clinical trials examining the effect of nut consumption on oxidative stress.

Reference	Subjects	Study Design/Period	Nut Type	Diet Intervention	Results
Durak et al., 1999 [77]	30 healthy subjects	Pre-post supplemental, dietary advice	Hazelnuts	1 g/kg body weight/day of hazelnuts supplemented with a habitual diet vs. baseline	↑ 0.5 kg body weight
					↓ TC (*p* < 0.005) , ↓ LDL-C (*p* < 0.0005), ↑ HDL-C, ↑ TG (*p*< 0.001), ↑ HDL-C/LDL-C, (*p* < 0.0005)
					↓ MDA(*p* < 0.0005), ↑ AOP (*p* < 0.0005) after hazelnut supplementation
Kocyigit et al., 2006 [71]	44 health subjects	Randomised parallel arm/3 weeks	Pistachios	20% energy of regular diet replaced with pistachio vs. regular diet	↔ body weight, ↔ energy intake
					↓ TC, ↓ MDA, ↓ TC/HDL-C (*p* < 0.001)
					↓ LDL-C/HDL-C (*p* < 0.01), ↑ HDL-C (*p* < 0.001), ↑ AOP, ↑ AOP/MDA
Jia et al., 2006 [205]	30 healthy adult male regular smokers	Parallel arm, control/4 weeks	Almonds	84 g/day almond group (*n* = 10) vs. 168 g/day almond group (*n* = 10) vs. Control group without nuts (*n* = 10)	↓ urinary 8-OH-dG, ↓ single strand DNA breaks,
					↓MDA, ↔ SOD, ↔ GSH-Px in almond groups compared with the control group
Haddad et al., 2006 [212]	24 healthy subjects	Randomized, controlled, crossover/4 weeks	Pecans	20% energy of a diet replaced with pecans vs. a control diet without nuts	↓ MDA, ↔ferric-reducing ability, ↔Trolox equivalent antioxidant capacity
Davis et al., 2007 [206]	65 subjects with metabolic syndrome	Randomised, parallel arm, control/8 weeks	Walnuts	20% energy of a habitual diet replaced with walnuts (63–108 g/day) vs. 20% energy of a habitual diet replaced with cashews (63–108 g/day) vs. Control diet without nuts	↓ GSSG, ↑ ORAC compared with baseline
Garg et al., 2007 [207]	17 male hypercholesterolemic subjects	Pre-post supplemental/4 weeks	Macadamias	15% energy of a habitual diet replaced with macadamias (40–90 g/day) vs. baseline	↓ oxidative status (8-isoprostane)
					↓ inflammation (leukotriene, LTB4)
					↔ TXB2/PGI2 ratio
Li et al., 2007 [208]	60 healthy male habitual smokers	Randomized, crossover/4 weeks	Almonds	Smokers; 84 g/day almonds vs. 120 g/day of pork (for smokers)	↑ serum a-tocopherol, ↑ SOD, ↑ GPX,
	30 healthy male				↔ caltalase after almond intake compared with no change in smokers after pork intake
	non-smokers			Non-smokers; 120 g/day of pork for reference comparison	↓ DNA strand breaks, ↓ 8-OHdG, ↓ MDA, in smokers after almond intake compared with baseline
Jenkins et al., 2008 [209]	27 hyperlipidemic subjects‘	Randomized, crossover/4 weeks	Almonds	73 g/day almonds added to self-selected low-fat therapeutic diet vs. 36.5 g/day almonds added to self-selected low-fat therapeutic diet vs. self-selected low-fat therapeutic diet without nuts (control)	↓ MDA, ↓ urinary isoprostane on 73 g/day almonds compared with control
					↔ α-or γ-tocopherol,
Thomson et al., 2008 [203]	59 healthy subjects	Randomized controlled/12 weeks	Brazil nuts	2 Brazil nuts (providing ≈100 μg Se, 100 μg Se as selenomethionine) vs. Placebo	↑ selenium (*p* < 0.0001), ↑ GPx (*p* < 0.001)
McKay et al., 2010 [213]	21 non-smoking man and postmenopausal women aged over 50 years	Randomized crossover/6 weeks	Walnuts	42 g/day walnuts vs. 21 g/day walnuts	↔ antioxidant activity (ORAC, ORAC with perchloric acid (pca) precipitation, FRAP and TAP), ↔ biomarkers of antioxidant status (total thiols, phenols, carotenoids and GPx), ↔ MDA
Lopez-Uriarte et al., 2010 [121]	50 subjects with metabolic syndrome	Randomised, controlled, parallel/4 weeks	Mixed nuts	30 g/day mix nuts (15 g walnuts, 7.5 g almonds and 7.5 g hazelnuts) vs. control diet without nuts	↓ DNA damage (*p* < 0.001),
					↔ Antioxidant capacity, ↔oxidized LDL, ↔ conjugated diene,
					↔ 8-iso-prostanes, ↔ endothelial function
Kay et al., 2010 [210]	28 hypercholesterolemic subjects	Randomized, crossover controlled/4 weeks	Pistachios	20% energy of a low-fat diet replaced with 63–126 g/day pistachios vs. 20% energy of a low-fat diet replaced with 32–63 g/day pistachios vs. a low-fat control diet without pistachios	↓ oxidized-LDL compared with a low-fat control diet
					↑ lutein (*p* < 0.0001), ↑ α-carotene, ↑ β-carotene (*p* < 0.01)compared with baseline
Sari et al., 2010 [24]	32 healthy young men	Dietary intervention/8 weeks	Pistachios	Subjects consumed a Mediterranean diet for 4 weeks and in the next 4 weeks they supplemented the Mediterranean diet with pistachios for 4 weeks replacing 32% of the energy obtained from MUFAs in the control diet (20% of energy)	↓ glucose levels (−8.8 ± 8.5% *p* < 0.001),
					↓ TC ( *p* < 0.001) ↓ LDL-C (*p* < 0.001), ↓ triacylglycerol, ↔ HDL-C, ↓ TC-C/HDL-C ↓ LDL-C/HDL-C ( *p* < 0.001),
					↑ endothelium-dependent vasodilation (*p* = 0.002)
					↓ lipid hydroperoxide (*p* < 0.001),
					↓ MDA (*p* < 0.001), ↓ IL-6 (*p* < 0.001),
					↑ SOD (*p* < 0.001), ↔ hs CRP, ↔ TNF-α
					compared with a Mediterranean diet
Haddad et al., 2014 [211]	healthy young subjects.	Randomized, crossover, controlled, acute 5-h test	Walnuts	A walnut meal containing 90 g walnuts and a isocaloric control meal (50% carbohydrate; 20% protein; 30% fat)	↓ oxidative stress, ↑ iAUC for hydrophilic and lipophilic ORAC, ↓ iAUC for MDA
					↓ oxidized LDL, ↑ epicatechin gallate
					↑ epicallocatechin gallate, ↑ urolithin-A in urine

AOP, antioxidant potential index; 8-OH-dG, urinary 8-hydroxy-2′-deoxyguanosine; FRAP, ferric reducing antioxidant power; GSH-Px, glutathione peroxidase; GSSG, oxidized glutathione; MDA, malondialdehyde; ORAC, oxygen radical absorbance capacity; PGI2, prostacyclin; SOD, superoxide dismutase; TAP, total antioxidant performance; TXB2, plasma thromboxane; ↑, increase; ↓, decrease; ↔, no effect.

**Table 5:** Summary of clinical trials examining the effect of nut consumption on inflammation.

Reference	Subjects	Study Design/Period	Nut Type	Diet Intervention	Results
Zhao et al., 2004 & 2007 [222,223]	23 hypercholesterolemic subjects	Randomized, controlled cross-over/6 weeks	Walnuts	Three diets; - AAD—13% energy from SFA, 13% energy from MUFAs and 8.7% energy from PUFAs (7.7% LA; 0.8% ALA) - The LA diet consisted of 16.4% energy from PUFAs (12.6% LA; 3.6% ALA) - The ALA diet consisted of 17% energy from PUFAs (10.5% LA; 6.5% ALA). Walnuts and walnut oil were used for half of total fat in the LA and ALA diets. The ratios of LA (*n*-6) to ALA (*n*-3) for the AAD, LA and ALA diet were 10:1, 4:1, and 2:1	↓ hs-CRP (*p* < 0.01) on ALA diet compared with the AAD
					↓ IL-6, ↓ IL-1β, ↓ TNF-α cultured in PBMCs on the ALA diet than on the LA diet
					↓ TC, ↓ TG, ↓ LDL-C ↓ ICAM-1 on both ALA and LA diets compared with AAD diet
					↓ VCAM-1 (*p* < 0.01), ↓ E-selectin (*p* < 0.01) on the ALA diet than on the LA diet
					↓ HDL-C and apolipoprotein AI on ALA diet compared with AAD diet
Garg et al., 2007 [207]	17 male hypercholesterolemic subjects	Pre-post supplemental/4 weeks	Macadamias	15% energy of a habitual diet replaced with macadamias (40–90 g/day) vs. baseline	↓ oxidative status (8-isoprostane)
					↓ inflammation (leukotriene, LTB4)
					↔ TXB2/PGI2 ratio
Rajaram et al., 2010 [224]	25 health subjects	Randomised, controlled, crossover/4 weeks	Almonds	High-almond diet (20% energy of a control diet replaced with almonds) vs. Low-almond diet (10% energy of a control diet replaced with almonds) vs. Heart-healthy control diet without nuts	↓ hs -CRP ↓ E-selectin (*p* < 0.0001)
Sari et al., 2010 [24]	32 healthy young men	Dietary intervention/8 weeks	Pistachios	Subjects consumed a Mediterranean diet for 4 weeks and in the next 4 weeks they supplemented the Mediterranean diet with pistachios for 4 weeks replacing 32% of the energy obtained from MUFAs in the control diet (20% of energy)	↓ glucose levels (−8.8 ± 8.5% *p* < 0.001)
					↓ TC, ↓ LDL-C, ↓ triacylglycerol, ↔ HDL-C, ↓ TC-C/HDL-C ↓ LDL-C/HDL-C
					↑ endothelium-dependent vasodilation
					↓ lipid hydroperoxide, ↓ MDA, ↓ IL-6,
					↑ SOD, ↔ hs CRP, ↔ TNF-α
					compared with a Mediterranean diet
Hernandez-Alonso et al., 2014 [225]	54 subjects with prediabetes	Randomized, controlled cross-over/4 months	Pistachios	A diet supplemented with 57 g/day pistachio vs. a control diet	↓ IL-6 mRNA, ↓ resistin gene expression
					↑ SLC2A4 expression
					↓ fasting glucose, ↓ insulin, ↓ HOMA-IR
					↓ fibrinogen, ↓ oxidized LDL, ↓ platelet factor 4
					↑ GLP-1
Sauder et al., 2015 [22]	30 subjects with well-controlled T2DM	Randomized, crossover, controlled/4 weeks	Pistachios	Healthy diet with pistachios contributing 20% of total energy. Low-fat or fat-free snacks (e.g., pretzels) in the control diet were substituted with roasted pistachios providing 20% of daily energy (59 to 128 g/day of pistachios). Half of amount of pistachio was ingested unsalted vs. AHA Therapeutic Lifestyle Changes diet (26.9% total fat, 6.7% SFA, 186 mg/day cholesterol).	↓ fructosamine, ↓ TC, ↓ ratio of TC to HDL-C, ↓ TG (*p* = 0.003), ↔ HbA1c,
					↔ fasting glucose, ↔ insulin, ↔ hs-CRP,
					↔ ICAM, ↔ VCAM, ↔endothelia function
Arpon et al., 2017 [221]	36 subjects at high cardiovascular risks	Randomised, controlled, parallel/5 years	Mixed nuts	Mediterranean diet supplemented with nuts (30 g/day of nuts) vs. Mediterranean diet supplemented with extra virgin olive oil (1 L/week of virgin olive oil) vs. Low-fat diet (control)	↑ association between methylation changes of 8 genes (EEF2, COL18A1, IL4I1, LEPR, PLAGL1, IFRD1, MAPKAPK2, and PPARGC1B) related to inflammation and Mediterranean diets ↑ association between EEF2 methylation and TNF-α and hs-CRP on Mediterranean diets compared with baselines.
Gulati et al., 2017 [226]	52 subjects with T2DM	Pre-post supplemental study, dietary advice, physical activity (45 min of walking at least 5 days some week)/24 weeks	Almonds	20% of total energy of a diet replaced with almonds vs. baseline	↓ hs-CRP (*p* < 0.01),
					↓ waist circumference,
					↓ waist-to-height ratio,
					↓ TC, ↓ TG, ↓ LDL-C, ↓ HbA1c

AAD, average American diet; ALA, α-linolenic acid; GLP-1, glucagon-like peptide-1; HbA1c, glycosylated haemoglobin; HDL-C, high density lipoprotein cholesterol; HOMA-IR, homeostasis model assessment of insulin resistance; hs-CRP, high sensitivity C-reactive protein; ICAM-1, intercellular cell adhesion molecule-1; LA, linoleic acid; LDL-C, low density lipoprotein cholesterol; MUFA, monounsaturated fatty acids; PBMC, peripheral blood mononuclear cell; PUFA, polyunsaturated fatty acids; TC, total cholesterol; TG, triglyceride; VCAM-1, vascular cell adhesion molecule-1; ↑, increase; ↓, decrease; ↔, no effect.
